# CSF hyperdynamics in rats mimicking the obesity and androgen excess characteristic of patients with idiopathic intracranial hypertension

**DOI:** 10.1186/s12987-024-00511-1

**Published:** 2024-01-25

**Authors:** Jonathan H. Wardman, Søren Norge Andreassen, Trine L. Toft-Bertelsen, Mette Nyholm Jensen, Jens E. Wilhjelm, Bjarne Styrishave, Steffen Hamann, Steffen Heegaard, Alexandra J. Sinclair, Nanna MacAulay

**Affiliations:** 1 Department of Neuroscience, University of Copenhagen, Blegdamsvej 3, Copenhagen, DK-2200 Denmark; 2 Department of Neurophysiology, Rigshospitalet, Copenhagen, Denmark; 3 Department of Health Technology, Technical University of Denmark, Copenhagen, Denmark; 4 Department of Pharmacy, University of Copenhagen, Copenhagen, Denmark; 5 Department of Ophthalmology, Rigshospitalet, Copenhagen, Denmark; 6 Department of Clinical Medicine, University of Copenhagen, Copenhagen, Denmark; 7 Institute of Metabolism and Systems Research, College of Medical and Dental Sciences, University of Birmingham, Birmingham, UK

**Keywords:** Choroid plexus, IIH, Cerebrospinal fluid, Testosterone, Sex hormones, Intracranial pressure, Transcriptomics

## Abstract

**Background:**

Idiopathic intracranial hypertension (IIH) is a syndrome exhibiting elevated intracranial pressure (ICP), visual disturbances, and severe headache. IIH primarily affects young obese women, though it can occur in individuals of any age, BMI, and sex. IIH is characterized by systemic metabolic dysregulation with a profile of increased androgen hormones. However, the contribution of obesity/hormonal perturbations to cerebrospinal fluid (CSF) dynamics remains unresolved.

**Methods:**

We employed obese female Zucker rats and adjuvant testosterone to reveal IIH causal drivers. ICP and CSF dynamics were determined with in vivo experimentation and magnetic resonance imaging, testosterone levels assessed with mass spectrometry, and choroid plexus function revealed with transcriptomics.

**Results:**

Obese rats had undisturbed CSF testosterone levels and no changes in ICP or CSF dynamics. Adjuvant testosterone treatment of obese rats elevated the CSF secretion rate, although with no effect on the ICP, due to elevated CSF drainage capacity of these rats.

**Conclusions:**

Obesity in itself therefore does not suffice to recapitulate the IIH symptoms in rats, but modulation of CSF dynamics appears with adjuvant testosterone treatment, which mimics the androgen excess observed in female IIH patients. Obesity-induced androgen dysregulation may thus contribute to the disease mechanism of IIH and could potentially serve as a future therapeutic target.

**Supplementary Information:**

The online version contains supplementary material available at 10.1186/s12987-024-00511-1.

## Introduction

Idiopathic intracranial hypertension (IIH) is a syndrome characterized by elevated intracranial pressure of an unknown origin resulting in disabling symptoms including headache, visual disruption due to optic nerve head swelling (papilledema), and cognitive impairment [1–6]. This syndrome almost exclusively affects obese women of child-bearing age, covering over 90% of all IIH cases [5–8]. With rapidly increasing global rates of obesity [9], incidence of IIH has also monumentally increased, with over 350% more cases in the past decade [7, 10–12]. Not only does increasing prevalence of obesity promise to increase IIH prevalence, but the increasing severity of obesity in the population will likely lead to increased prevalence of IIH, and associated increased severity of symptoms, since these correlate with elevated BMI [9]. While avoidance of permanent visual impairment has improved with increased awareness and early intervention to prevent the most severe symptoms [5–7], disability can still arise from headache and cognitive impairment symptomatic of IIH [3, 8, 10]. The definitive intervention to manage IIH is weight loss, either via diet or bariatric surgery [13], but acute surgical intervention is required in cases where vision is threatened [6]. Reduction of ICP can be obtained with alleviation of the CSF burden via ventriculoperitoneal shunt, which diverts CSF from the brain ventricles to the abdomen [2, 13, 14] or, alternatively, via pharmacological treatment using either acetazolamide or topiramate, which lowers CSF production [2, 15]. However, potential complications and need for revision of surgical procedures [2, 6], and low adherence to pharmacological treatments due to severe side effects [2], obviate a need for more effective treatments with fewer complications. As both interventions target reduction of CSF volumes, a better understanding of the contribution of CSF production to IIH etiology could lead to effective strategies to treat elevated ICP in IIH. The elevated ICP that is so characteristic of IIH has previously been attributed to aspects of CSF dynamics including elevated venous pressure [16, 17], increased CSF production [18–20], reduced CSF drainage capacity [20–23], and increased brain fluid content [24–26], possibly related to venous sinus stenosis [27, 28], or changes in brain tissue compliance due to fibrogenesis and basement membrane deterioration [29]. However, these proposed contributors to IIH etiology have been contested [30–32] and changes in CSF dynamics in IIH patients are therefore yet to be satisfactorily assessed. The invasive nature of some CSF measurement techniques prevents the employment of IIH patients for thorough investigation of these aspects. An experimental animal model recapitulating IIH symptomatology would allow a complete characterization of the contributions from variables involved in CSF dynamics. To this end, previous studies have employed high-fat diet (HFD) to induce obesity in female rodents with demonstration of some degree of elevated ICP in these rats, in part due to their reduced CSF drainage capacity [33, 34], although with no obvious changes to CSF flow rates [34].

It is unsurprising that appropriate age, obesity, and female sex alone are unable to completely recapitulate IIH in rodents. Among women fitting this description, i.e. young and obese, the incidence of IIH is only 15.2/100,000 [7], indicating that factors other than merely obesity are required for IIH manifestation. Among many other putative factors contributing to IIH etiology, such as altered levels of hormones, inflammatory markers, and enzyme activity [35–37], most notable is androgen (testosterone) excess in serum and CSF of IIH patients, differentiating them from unaffected obese females [36]. Notably, treatment of lean female rats with adjuvant testosterone leads to increased CSF production and elevated ICP, in part due to elevated activity of the choroid plexus Na^+^,K^+^,2Cl^−^ cotransporter NKCC1 [34] which is a key contributor to CSF production [38–41], suggesting a possible connection between IIH-related testosterone burden and disturbed CSF dynamics.

Neither obesity nor androgen levels can be readily manipulated in patient populations to determine their contributions to IIH etiology. High-fat diet animal models have not reached obesity levels fully recapitulating those observed in the IIH patient group [33, 34], indicating that an updated animal model combining adequate obesity and adjuvant testosterone might better illustrate the potential contribution of altered CSF dynamics to IIH symptoms. To this end, we have employed genetically obese female Zucker rats with and without adjuvant testosterone to determine the impact of a combination of these factors in IIH etiology and symptomatology, including ICP, brain fluid dynamics, choroid plexus function, and ventricular morphology.

## Methods

### Experimental animals

Animal handling and experiments were performed according to European guidelines and complied with all ethical regulations. All experiments were approved by the Danish Animal Experiments Inspectorate with permission no. 2018-15-0201-01515. Female heterozygous lean (*+/fa*) and obese (*fa/*fa) Zucker rats (Charles River Laboratories), were received aged 6 weeks, and maintained on normal chow diet for 10 weeks prior to experimentation. Diet and water were provided *ad libitum* and the rat weights recorded on a weekly basis. For testosterone experiments, female obese Zucker rats were injected twice-weekly with 1 mg testosterone propionate in 100 µl sesame oil (86,541, Sigma-Aldrich) [42] and female lean Zucker rats were injected with 100 µl sesame oil (S3547, Sigma-Aldrich, vehicle control) subcutaneously starting four weeks prior to experimentation. Testosterone administration was calculated to obtain supraphysiological testosterone levels concomitant with those observed in female IIH patients [36, 42].

### Anesthesia and ventilation

Anesthesia was predominantly implemented via intraperitoneal (i.p.) injection of 6 mg/ml xylazine + 60 mg/ml ketamine (ScanVet) in sterile water (0.17 ml/100 g bodyweight, pre-heated to 37° C). Animals were re-administered half ketamine dose as required to sustain anesthesia. One rat was excluded because it was unresponsive to initial anesthesia administration. The body temperature was maintained at 37 °C by a homeothermic monitoring system (Harvard Apparatus). Mechanical ventilation was employed for all anesthetic protocols lasting more than 30 min, to ensure stable respiratory partial pressure of carbon dioxide (pCO_2_) and oxygen (pO_2_) and arterial oxygen saturation and thus stable plasma pH and electrolyte concentration. Surgical tracheotomy was carried out for mechanical ventilation, which was controlled by the VentElite system (Harvard Apparatus) by 0.9 l/min humidified air mixed with 0.1 l/min oxygen adjusted with approximately 2.6 ml per breath, 80 breath/min, a Positive End-Expiratory Pressure (PEEP) at 2 cm, and 10% sight for both lean and obese rats. Ventilation settings were optimized for each animal using a capnograph (Type 340, Harvard Apparatus) and a pulse oximeter (MouseOx® Plus, Starr Life Sciences) after system calibration with respiratory pCO_2_ (4.5–5.0 kPa) and pO_2_ (13.3–17.3 kPa) and arterial oxygen saturation (98.8–99.4%) (ABL90, Radiometer).

### ICP and resistance to outflow (*R*_*out*_) measurements

Anesthetized and ventilated rats, placed in a stereotactic frame had the skull exposed, and were first implanted with a cannula (Brain infusion kit 2, Alzet) into the left lateral ventricle as described below in the ventriculo-cisternal perfusion section. After cementing the cannula in place, a 3.6 mm diameter cranial window was drilled with care not to damage the dura. An epidural probe (PlasticsOne, C313G) was secured with dental resin cement (Panavia SA Cement, Kuraray Noritake Dental Inc.) above the dura and the ICP probe was filled with HEPES-aCSF (in mM) 120 NaCl, 2.5 KCl, 2.5 CaCl_2_, 1.3 MgSO_4_, 1 NaH_2_PO_4_, 10 glucose, 17 Na-HEPES, adjusted to pH 7.4 with NaOH) before connection to a pressure transducer APT300 and transducer amplifier module TAM-A (Hugo Sachs Elektronik). To ensure the presence of a continuous fluid column between the dura and the epidural probe, approximately 5 µl HEPES-aCSF was initially injected through the epidural probe. The ICP signal was recorded at a 1 kHz sampling rate using BDAS Basic Data Acquisition Software (Hugo Sachs Elektronik). Jugular compression was applied to confirm proper ICP recording. Five rats in each group were employed to measure the ICP. After 30 min of ICP recording, resistance to CSF drainage assay was performed by pumping aCSF into the lateral ventricle cannula for intervals of 10 min at 5, 10, 15, and 20 µl/min, with a one minute pause between each rate increase. Resistance to drainage (*R*_*out*_) was calculated using the following equation (derived from [43]):

$$ {R}_{out}=\frac{{ICP}_{f}-{ICP}_{i}}{I}$$

where *ICP*_*f*_ = stable ICP after 10 min of infusion at a given rate in mmHg, *ICP*_*i*_ = initial, stable baseline ICP in mmHg, and *I* = infusion rate in µl/min. *R*_*out*_ for each rate of infusion was calculated and averaged across all infusion rates for each rat to determine resistance to CSF drainage. Six rats in each group were employed to measure the R_out_.

### ICP waveform analysis

The raw ICP data stored as semicolon separated values in normal text files by the BDAS software were read into MATLAB (MathWorks) and possible spikes and non-physiological data points removed. This was followed by low-pass filtering (anti-aliasing) and down sampling to 100 Hz. These signals were of length about 300 to 900 s and contained approximately 1.3 Hz frequency components from the artificial ventilation, the heart rate as well as other minor interferences. Since the heart rate was in the region 2.5 to 5 Hz, frequency components from the signal was spectrally bandpass filtered between 1.6 and 5.5 Hz with a Tukey window. The instantaneous peak-to-peak amplitude was finally extracted from the filtered time signals. The average of this is denoted mean wave amplitude (MWA). Five rats in each group were employed to quantify the ICP waveform.

### Retinal nerve fiber layer thickness analysis at the optic nerve head

The eyeballs were removed from the rats after anaesthetization and decapitation. The eyes were fixed in 4% buffered formaldehyde and embedded in paraffin. Sections were cut at 4 μm and mounted on glass slides. The sections were stained with hematoxylin-eosin (HE) and imaged. The optic nerve heads in both groups appeared normal without any pathology or signs of papilledema. For analysis of the retinal nerve fiber layer (RNFL) thickness, the best possible quality histological section from each rat covering the central portion of the optic nerve head, were used for measuring the RNFL thickness at the boundary between the retina and the optic nerve on each side of the optic nerve head using ImageJ commercial freeware (available at https://imagej.nih.gov/ij/download.html), as previously described [33, 44]. Six obese and testosterone-treated rats and three lean rats were employed to measure the RNFL thickness.

### Ventriculo-cisternal perfusion

Rats were anesthetized, ventilated, and an infusion cannula (Brain infusion kit 2, Alzet) was stereotactically placed in the right lateral ventricle. A 0.5 mm (diameter) burr hole was drilled (1.3 mm posterior, 1.8 mm lateral to bregma), and a 4 mm (length) brain infusion cannula (Brain infusion kit2, Alzet) was glued in place on the cranium with the cannula placed into the lateral ventricle, through which a pre-heated (37 °C, SF-28, Warner Instruments) $\text{HCO}_{3}^{-}$buffered aCSF ($\text{HCO}_{3}^{-}$-aCSF; (in mM) 120 NaCl, 2.5 KCl, 2.5 CaCl_2_, 1.3 MgSO_4_, 1 NaH_2_PO_4_, 10 glucose, 25 NaHCO_3_, equilibrated with 95% O_2_/5% CO_2_ to obtain a pH of 7.4) containing 1 mg/ml TRITC-dextran (tetramethylrhodamine isothiocyanate-dextran, MW = 150,000; T1287, Sigma), 307 mOsm, was infused at 9 µl/min. CSF was sampled from cisterna magna at 5 min intervals with a glass capillary (30–0067, Harvard Apparatus pulled by a Brown Micropipette puller, Model P-97, Sutter Instruments) placed at a 5° angle (7.5 mm distal to the occipital bone and 1.5 mm lateral to the muscle-midline). The cisterna magna puncture and continuous fluid sampling prevent elevation of ICP during the procedure. The fluorescent content of CSF outflow was measured in triplicate on a microplate photometer (545 nm, SyneryTM Neo2 Multi-mode Microplate Reader; BioTek Instruments), and the CSF secretion rate *V*_*p*_ (µl/min), was calculated from the equation [45]:

$$ {V}_{p}={r}_{i}\times \frac{{C}_{i}-{C}_{o}}{{C}_{o}}$$

where *r*_*i*_ = infusion rate (µl/min), *C*_*i*_ = fluorescence of inflow solution, *C*_*o*_ = fluorescence of outflow solution. Six obese rats and four lean rats were employed for ventriculo-cisternal perfusion.

### Live imaging of CSF movement

Rats were anesthetized, placed in a stereotactic frame and through a burr hole in the lateral ventricle (same coordinates as for ICP and ventriculo-cisternal perfusion) a Hamilton syringe (RN 0.40, G27, a20, Agntho’s) was placed (4 mm deep) with 15 µl $\text{HCO}_{3}^{-}$-aCSF with 10 µM carboxylate dye (MW = 1,091 Da, IRDye 800 CW, P/N 929–08972, LI-COR Biosciences). Immediately after the dye injection, the rat was swiftly placed in a Pearl Trilogy Small Animal Imaging System (LI-COR Biosciences) and within 1 min after ventricular dye injection, images were obtained at 30 s intervals (800 nm channel, 85 μm resolution, for 5 min). A white field image was acquired at the termination of each experiment, after which the rat was sacrificed. The isolated brain was then bisected to expose the ventricles to record a final micrograph ensuring proper targeting of the ventricular compartment. Images were analyzed in a blinded fashion using LI-COR Image Studio 5.2 (LI-COR Biosciences) and data presented as fluorescence intensity in a region of interest placed in line with lambda, normalized to the signal obtained in the first image. Five rats in each group were employed for the live-imaging of CSF flow.

### Brain water quantification

For brain water determination, rats were decapitated under anesthesia. The brain was rapidly dissected into a pre-weighed porcelain evaporating beaker (Witeg) and weighed within 1 min after brain isolation. The brain was then homogenized with a spatula (to increase surface area) and left at 100^°^C for approximately 90 h to dry. After drying, the brain was weighed again and the difference in the two measurements corresponded to the brain water (in g) and was employed to obtain the water percentage. Four lean rats and five obese rats were employed for brain water quantification.

### MRI analysis

Isoflurane-anesthetized rats underwent MRI in a 9.4 Tesla preclinical horizontal bore scanner (BioSpec 94/30 USR, Bruker BioSpin) equipped with a 240 mT/m gradient coil (BGA-12 S, Bruker) at the Preclinical MRI Core Facility, University of Copenhagen. The scanner was interfaced to a Bruker Avance III console and controlled by Paravision 6.1 software (Bruker). Imaging was performed with an 86 mm-inner-diameter volume resonator and a 4-channel surface quadrature array receiver coil. The animal body temperature was maintained at 37 ± 0.5 °C with a thermostatically controlled waterbed and its respiratory rate monitored by an MR-compatible monitoring system (SA Instruments). The imaging protocol consisted of T_2_-weighted 2D rapid acquisition with relaxation enhancement (2D-RARE) for reference spatial planning with the following settings: repetition time (TR) = 4000 ms, effective echo time (TE) = 60 ms, number of averaging (NA) = 4, RareFactor = 4, slice thickness = 500 μm, in-plane spatial resolution size = 137 × 273 μm, 25 coronal slices, total acquisition time (TA) = 8.5 min. For obtaining high resolution CSF volumetry, a 3D constructive interference steady-state sequence (3D-CISS) [46, 47] image was calculated as a maximum intensity projection (MIP) from 4 realigned 3D-TrueFISP volumes with 4 orthogonal phase encoding directions (TR = 4.6 ms, TE = 2.3 ms, NA = 1, Repetitions = 2, Flip angle = 50°, 3D spatial resolution 100 × 100 × 100 μm, RF phase advance 0, 180, 90, 270°, TA = 28 min). To obtain optimal spatial uniformity, all acquired 3D-TrueFISP volumes were motion-corrected before calculation as MIP, and the image bias field was removed with Advanced Normalization Tools (ANTs) [48, 49]. For each brain sample, the total brain volume was automatically segmented by using region growing with ITK-snap (version 3.8.0) [50]. In addition, the pixel intensity factorized semi-automatic thresholding was performed to segment the lateral ventricle in each hemisphere. The volume measurement of the whole brain and lateral ventricles were performed in ITK-snap. The analysis was carried out in a blinded fashion. Four rats in each group were employed for MRI.

### RNA sequencing

Choroid plexus (lateral and 4^th^) was rapidly isolated from rats after anaesthetization and decapitation; brains were submerged in cold HEPES-buffered aCSF for isolation and stored in RNAlater® (Sigma) at -80 °C prior to RNA extraction and library preparation with NEB Next® Ultra™ RNA Library Prep Kit (NEB) by Novogene. RNA sequencing (paired-end 150 bp, with 12 Gb output) was performed on an Illumina NovaSeq 6000 (Illumina). All program parameter settings for library building and mapping, together with all scripts for the gene annotation and analysis are available at https://github.com/Sorennorge/MacAulayLab-RNAseq3-Zucker. Raw data are available at the National Center for Biotechnology Information (NCBI) Gene Expression Omnibus (GEO) database (GSE232814). The sequencing data of 150 base paired-end reads were mapped to reference genome (Rattus norvegicus Rnor_6.0 v.104) using Spliced Transcripts Alignment to a Reference (STAR) RNA-seq aligner (v. 2.7.9a) [51]. The mapped alignment by STAR was converted to raw counts from STAR GeneCounts. The raw counts from STAR GeneCount were used for differential expression analysis using R library and program DEseq2 [52]. Differentially expressed genes were determined based on standard procedure of DEseq2 analysis with false discovery rate (FDR, Benjamini–Hochberg method) [53] of less than 0.05 [54]. The Volcano plot was created using R library ggplot2 [55], the subplot for the pie chart was created using python library matplotlib, and the subplot for Heatmap was generated using R library pheatmap [56]. The Gene Ontology (GO) enrichment analysis was generated utilizing the Panther database [57] with the gene symbols from the differentially expressed genes from DEseq2 to classify the protein class of each gene and the pie chart of the GO enrichment analysis created using python library matplotlib. Transporters from the enrichment analysis were extracted and compartment scores were calculated and only transporters with a ‘plasma membrane’ score above 2 were used for the bar chart [58]. The network analysis was generated from differentially expressed genes from DEseq2 using the gene symbols as protein database query from String-database (https://string-db.org/) [59] and only including connections with a string confidence score above 0.7 as a plugin for Cytoscape (v. 3.9.1) [60]. The node size is divided into 4 different categories according to the expression levels of the genes (less than one TPM, between 1 and 10 TPM, between 10 and 100 TPM, and more than 100 TPM). Network analysis, in respect to transporters of interest, was first generated with all first order connections to the transporters with the string DB with connection score above 0.7. Hereafter, the connections to differentially expressed genes were added. Six (lean vs. obese) or four (lean vs. obese and testosterone-treated) rats in each group were employed for RNAseq.

### Androgen quantification in CSF

Rats were anesthetized, placed in a stereotactic frame and CSF was extracted through a cisterna magna puncture and immediately centrifuged to pellet cell debris (2000 × *g*, 10 min, 4 °C) prior to storage of the ∼ 100 µl supernatant at -80 °C in sealed microcentrifuge tubes. The steroid extraction was performed as earlier described [61] with a further modification of the CSF analysis to encompass a different size of solid-phase extraction columns (100 mg Bond elute C_18_ solid-phase extraction cartridges; 1 ml; Agilent, USA) and therefore corresponding volume changes for conditioning (1 ml MeOH followed by 2 × 1 ml dH_2_O), washing (2 × 1 ml dH_2_O followed by 1 ml H_2_O:MeOH (3:1)) and elution (1 ml H_2_O:MeOH (1:4)). The liquid chromatography online clean-up, chromatographic separation of androgens and mass spectrometry data analysis were done as previously described [61]. Four rats in each group were employed for androgen quantification, but one statistically significant outlier was removed from the lean rat group and one from the obese and testosterone-treated group.

### Statistical analysis

Data analysis and statistical tests were carried out using Graphpad Prism version 9. For statistical analysis, student’s unpaired two-sample t-test was used for comparison of two groups of animals and linear regression was performed to detect potential correlation effects. The significance of the data was estimated based on a p-value < 0.05 and displayed with the mean and standard error of the mean (SEM).

## Results

### Obesity does not associate with increased intracranial pressure

**Fig. 1:**
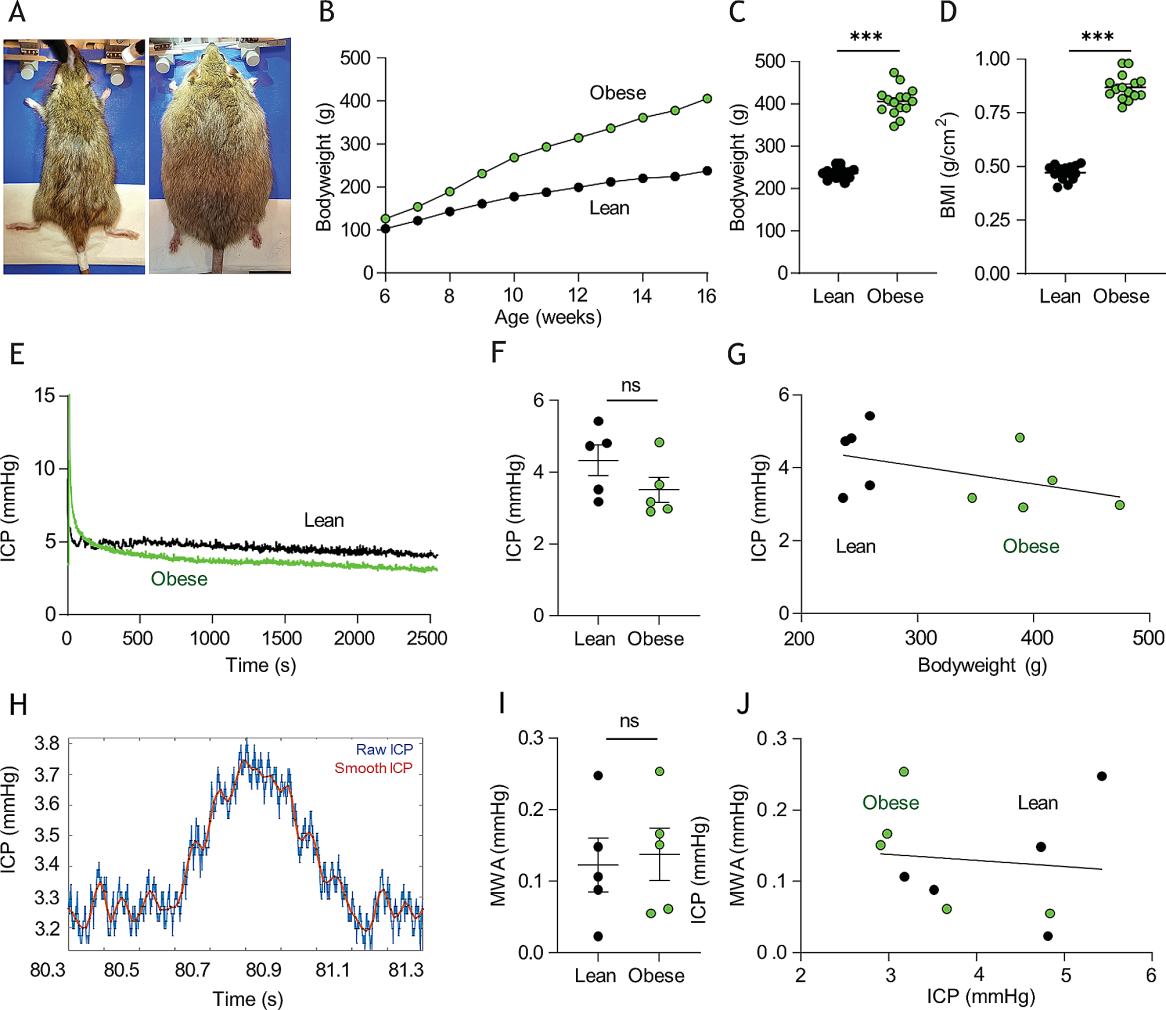
Zucker rat bodyweight and intracranial pressure. **A** Images of lean (left panel) and obese (right panel) Zucker rats. **B** Bodyweight increases as a function of time of Zucker lean (n = 15) and obese (n = 16) rats included in study. Error bars are within the symbols. **C** Rat bodyweight and **D** body mass index (BMI) at time of experimentation. **E** representative intracranial pressure (ICP) traces from Zucker lean and obese rats with the final 15 min recordings quantified in **F**, n = 5 of each. **G** Correlation analysis of ICP as a function of bodyweight in the tested Zucker rats. **H** Representative ICP trace with “raw” (blue line) and “smoothed” (red line) signals, the latter used to calculate the mean wave amplitude (MWA), represented in **I** (n = 5 of each). **J** Correlation analysis of MWA as a function of ICP, n = 10. Statistical significance evaluated with Student’s unpaired t-test or simple linear regression and results shown as mean ± SEM. ***p < 0.001, ns = not significant

To obtain an animal model representative of the obesity characteristic of the majority of IIH patients, we employed a female rat model of obesity originating from a genetic deficiency in the leptin receptor. These Zucker rats then do not experience satiation and become obese due to hyperphagia of a normal chow diet [62], Fig. 1A. At the time of experimentation (16 weeks of age), the obese Zucker rats (henceforward termed ‘obese’) weighed 406 ± 9 g, *n* = 15 vs. their non-mutated lean counterpart (henceforward termed ‘lean’) at 236 ± 3 g, *n* = 16, *p* < 0.001, Fig. 1B-C, with rodent body mass indexes (BMI) of 0.87 ± 0.02 g/cm^2^ in the obese rats, *n* = 15 vs. 0.47 ± 0.01 g/cm^2^ in the lean rats, *n* = 16, *p* < 0.001 (Fig. 1D). Despite the severe obesity of the experimental rat cohort, their ICP (3.51 ± 0.36mmHg, *n* = 5) was not significantly different from their lean counterparts (4.33 ± 0.42 mmHg, *n* = 5, *p* = 0.18, Fig. 1E-F) with no correlation between ICP and bodyweight (*n* = 10, R^2^ = 0.21, *p* = 0.18, Fig. 1G). ICP waveform analysis revealed similar mean wave amplitudes in the two rodent cohorts (0.14 ± 0.04 mmHg, *n* = 5 in the obese rats vs. 0.12 ± 0.04 mmHg, *n* = 5 in the lean rats, *p* = 0.78, Fig. 1H-I) and showed no significant correlation with ICP (*n* = 10, R^2^ = 0.01, *p* = 0.78, Fig. 1J). The obese Zucker rats therefore did not present with the elevated ICP characteristic of IIH patients.

### Obesity in itself does not influence the CSF secretion rate

**Fig. 2:**
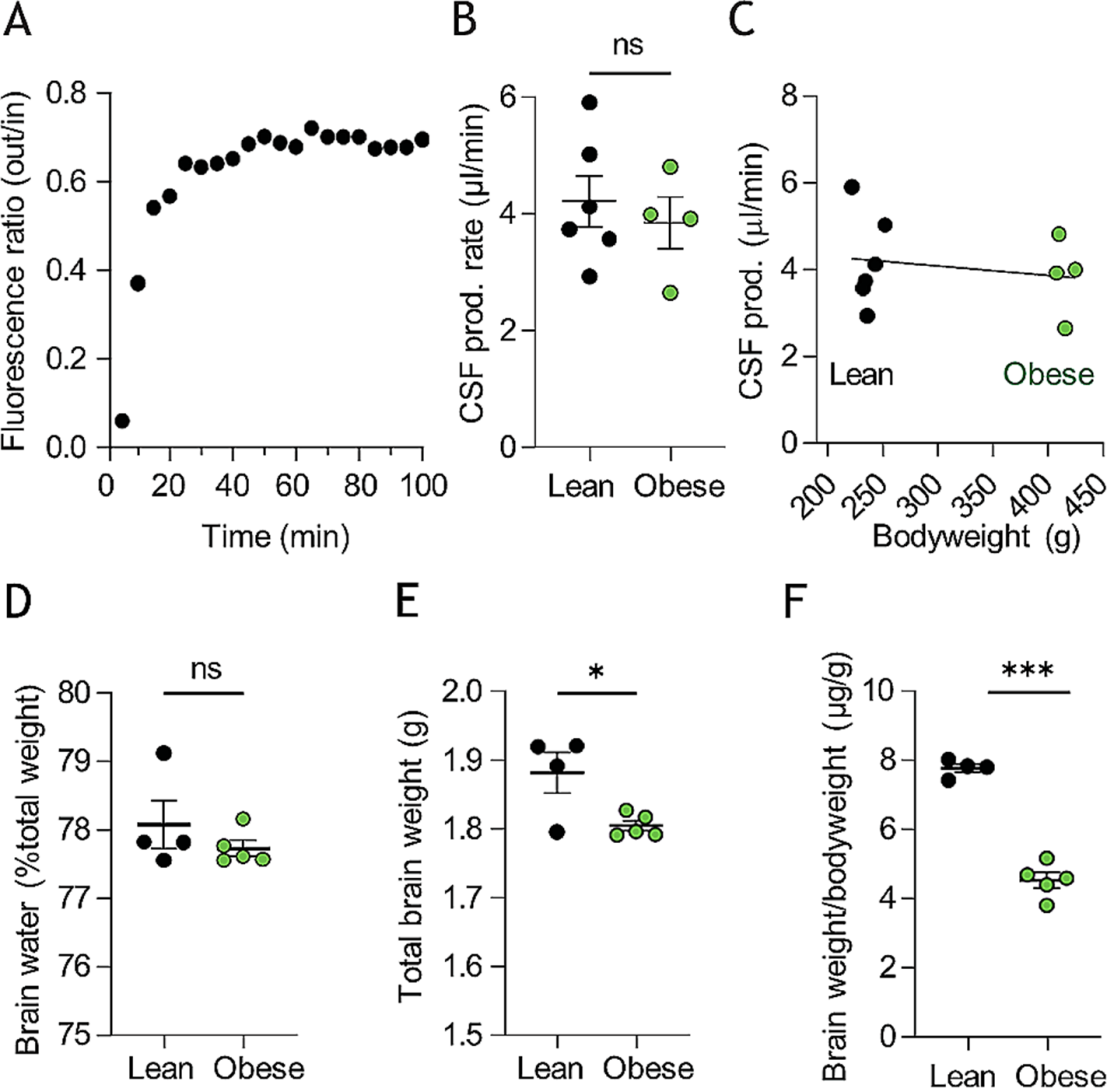
Zucker rats CSF production rates and brain water content. **A** Representative trace of fluorescent dye dilution over the course of a ventriculo-cisternal perfusion assay in a Zucker lean rat with the final 30 min employed for quantification of the CSF production rate illustrated in **B**, *n* = 4–6. **C** illustrates the CSF production rate as a function of bodyweight, with no significant correlation between the two parameters. **D** percentage brain water content in Zucker lean and obese rats, *n* = 4–5. **E** Brain weight and **F** relative brain weight of lean and obese Zucker rats, *n* = 4–5. Statistical significance evaluated with Student’s unpaired t-test or simple linear regression and results shown as mean ± SEM. *p < 0.05, ***p < 0.001, ns = not significant

To determine whether rodent obesity modulates the CSF dynamics, we determined the CSF secretion rate in vivo with the ventriculo-cisternal perfusion technique [38]. Here, fluorescent dextran dissolved in heated and gas-equilibrated artificial cerebrospinal fluid (aCSF) is delivered continuously into the lateral ventricle of anesthetized and mechanically ventilated rats with concomitant fluid collection from a cisterna magna puncture. During fluid passage through the ventricles, the dextran is diluted with the endogenously secreted CSF, the ratio of which is employed to calculate the rate of CSF production (Fig. 2A). The CSF secretion rate was similar in the two cohorts (3.84 ± 0.89 µl/min, *n* = 4 in the obese rats vs. 4.22 ± 1.08 µl/min, *n* = 6 in the lean rats, *p* = 0.58, Fig. 2B) and did not display correlation with bodyweight (*n* = 10, R^2^ = 0.02, *p* = 0.64, Fig. 2C). Accordingly, the brain water percentage remained comparable in the two cohorts (77.7 ± 0.3% in the obese rats, *n* = 5 vs. 78.1 ± 0.7% in the lean rats, *n* = 4, *p* = 0.34, Fig. 2D), although the brain mass was significantly lower in the obese rats, whether determined as total brain weight (1.81 ± 0.02 g, *n* = 5 in the obese rats vs. 1.88 ± 0.06 g, *n* = 4 in the lean rats, *p* < 0.05, Fig. 2E) or relative to bodyweight (4.52 ± 0.22 µg/g, *n* = 5 in the obese rats vs. 7.76 ± 0.12 µg/g, *n* = 4 in the lean rats, *p* < 0.001, Fig. 2F).

### Undisturbed brain fluid distribution with obesity

**Fig. 3:**
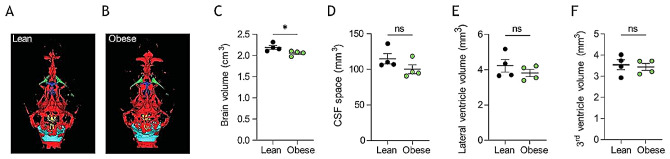
MRI analysis of CSF spaces in Zucker rats. **A-B** Representative T_2_ MRI images from a lean (**A**) and an obese (**B**) Zucker rats displaying total CSF spaces illustrated in green (lateral ventricles), dark blue (3^rd^ ventricle), yellow (4^th^ ventricle) or light blue (cisterna magna). **C** Brain volume obtained from the MRI scans, *n* = 4 of each. **D** Total CSF space, **E** Lateral ventricle volume, and **F** 3^rd^ ventricle volume obtained from the MRI scans, *n* = 4 of each. Statistical significance evaluated with Student’s unpaired t-test and results shown as mean ± SEM. *p < 0.05, ns = not significant

To elucidate whether the brain water dispersed differentially with obesity, we performed MRI analysis on the two rodent cohorts (Fig. 3A-B). Initial analysis demonstrated a slightly reduced brain volume in the obese Zucker rats (2091 ± 31 mm^3^, *n* = 4 in the obese rats vs. 2244 ± 47 mm^3^, *n* = 4 in the lean rats, *p* < 0.05, Fig. 3C), which aligns with the lower brain mass (see Fig. 2E-F). However, despite the overall smaller brain volume, the total CSF space was similar in the two groups (100 ± 6 mm^3^ in obese rats vs. 115 ± 7 mm^3^ in lean rats, *n* = 4 of each, *p* = 0.17, Fig. 3A-B,D), as was the lateral ventricle volume (3.81 ± 0.21 mm^3^ in obese rats vs. 4.22 ± 0.35 mm^3^ in lean rats, *n* = 4 of each, *p* = 0.35, Fig. 3E), and the third ventricle volume (3.44 ± 0.16 mm^3^ in obese rats vs. 3.54 ± 0.23 mm^3^ in lean rats, *n* = 4 of each, *p* = 0.73, Fig. 3F, see Additional file 1 for quantification of all CSF spaces). The data suggest that the CSF fluid distribution, a reflection of CSF dynamics, remains stable with obesity.

### Transcriptional changes in choroid plexus with obesity

**Fig. 4:**
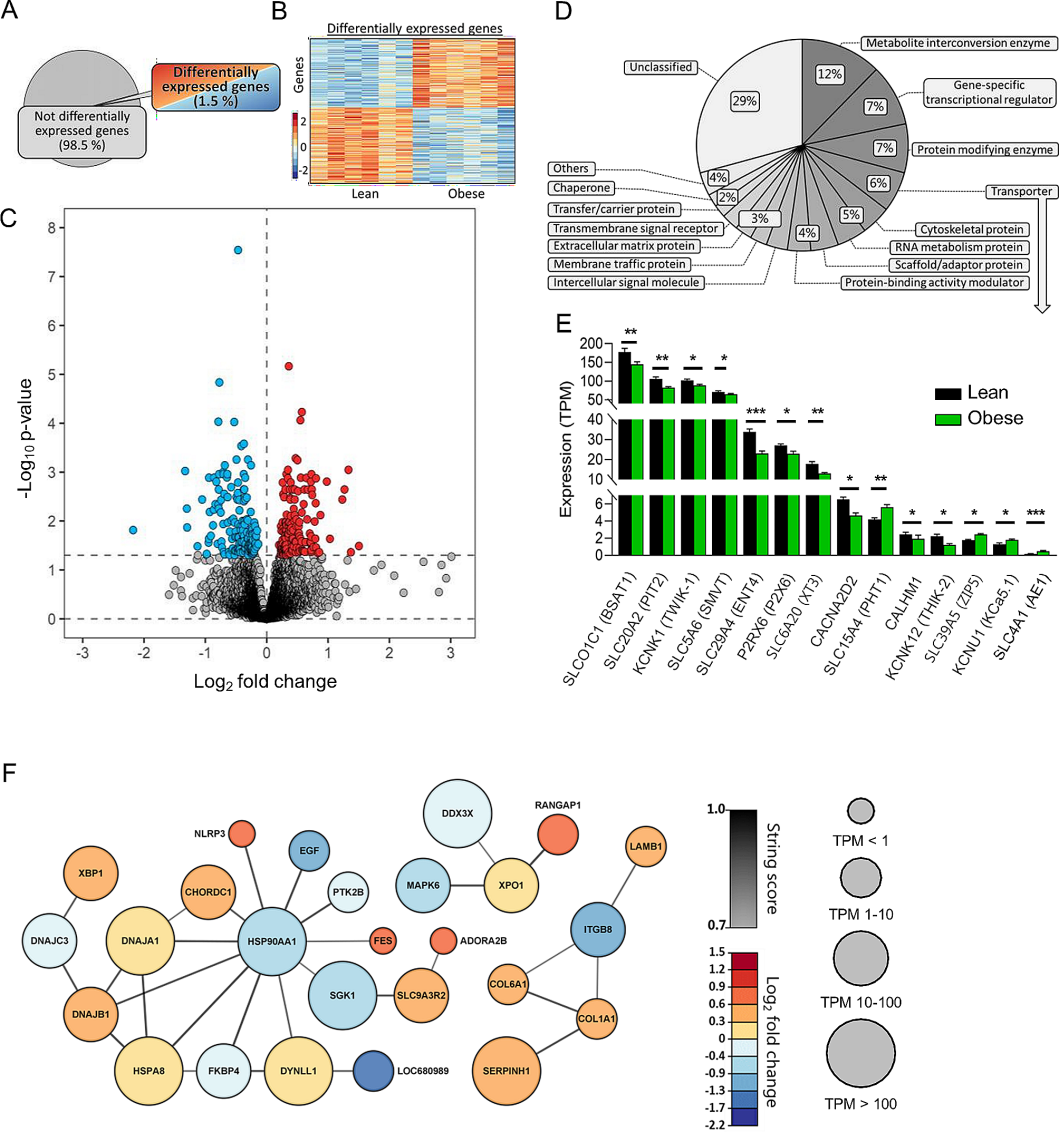
RNA expression in Zucker obese rats. **A** illustrates a pie chart of the percentage differentially expressed genes. **B** A heatmap of the differentially expressed genes in the individual Zucker rats, coloured based on the Z-score of normalized fold changes (*n* = 6 of each). **C** Volcano plot of all transcribed genes detected in the choroid plexus of lean and obese Zucker rats with differentially expressed genes (*p* < 0.05) marked in red (upregulated) or blue (downregulated). **D** GO enrichment analysis of the protein class categories. **E** Bar chart of all differentially expressed transporters in lean vs. obese Zucker rats. Bar height is expression levels in transcript per million (TPM) with significance levels (* *p* < 0.05, ** *p* < 0.01, and *** *p* < 0.001). **F** Association protein network analysis of the highest order cluster of genes. The protein nodes are coloured based on log2FC (downregulated in blue) and the connecting lines coloured based on the string confidence score from 0.7 (grey) to 1.0 (black). Size of the nodes are based on expression levels in four different categories

The choroid plexus is the principal source of CSF secretion and obesity-dependent modulation of its cellular and molecular components could influence brain fluid dynamics in numerous manners. To obtain an unbiased characterization of the choroid plexus transcriptomic profile of obese and lean Zucker rats, we performed RNAseq analysis on choroid plexus tissue extracted from these animals. Of the 21,252 expressed genes detected in the Zucker rat choroid plexus (Additional file 2, Sheet 1), 316 genes (1.5%) were differentially expressed (Fig. 4A). A heatmap illustrated approximately evenly distributed up- and downregulated genes (Fig. 4B), with a Volcano plot demonstrating 151 upregulated genes and 165 downregulated genes (Fig. 4C and Additional file 2, Sheet 2). We employed GO enrichment analysis of the 316 differentially expressed genes, where 218 genes could be categorized in protein classes to reveal the functional categories of the differentially expressed genes. These transcribed genes were dispersed in 14 different protein categories, including a pool of ‘others’ containing 10 categories with less than 4 genes assigned to these, and a pool of genes not assigned to any protein category, labelled ‘unclassified’ (Fig. 4D). The most abundant categories include metabolite interconversion enzymes (12%), gene-specific transcriptional regulators (9%), protein-modifying enzymes (7%), and transporters (6%). With the latter potentially able to directly influence CSF secretion, we extracted the differentially expressed plasma membrane transporters within this category and illustrated their transcriptional abundance in Fig. 4E (Additional file 2, Sheet 3). The percentage of obesity-induced transcriptional changes was generally negligible, and the transcript levels mostly downregulated with obesity (Fig. 4E and Additional file 2, Sheet 3). Taken together with the limited network-associated transcript regulation (Fig. 4F), the overall choroid plexus transcriptomic profile provides no direct indication of an obesity-related alteration in the CSF secretion machinery that could contribute to obesity-mediated disturbances in CSF dynamics.

### Adjuvant testosterone does not influence the obese Zucker rat ICP

**Fig. 5:**
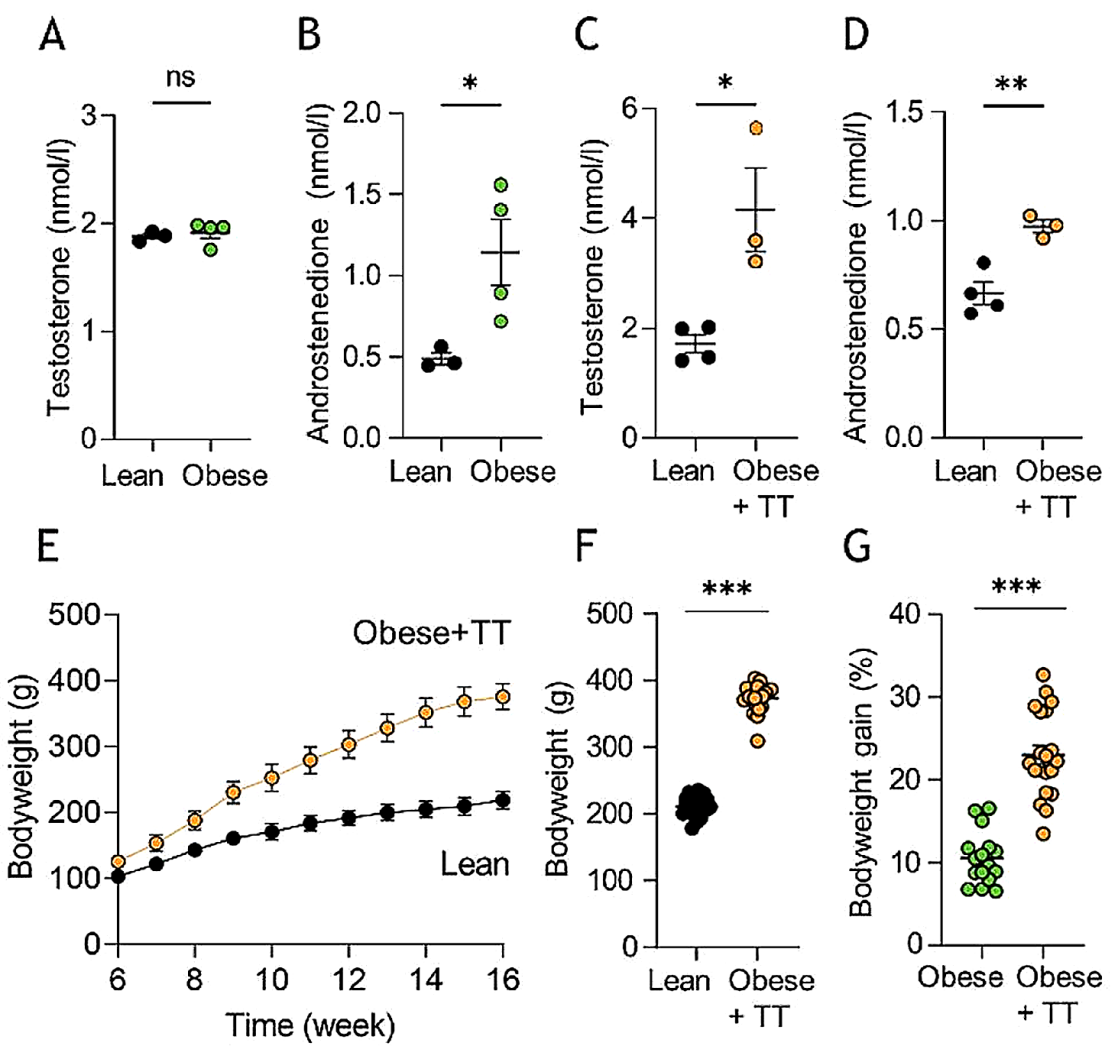
CSF androgen analysis and testosterone treatment of Zucker rats. **A** CSF testosterone levels in lean and obese Zucker rats, *n* = 3–4. **B** CSF androstenedione levels in lean and obese Zucker rats, *n* = 3–4. **C** CSF testosterone levels in lean and testosterone-treated obese (four weeks, obese + TT) Zucker rats, *n* = 3–4 (after one outlier removed from the obese + TT group). **D** CSF androstenedione levels in lean and obese + TT Zucker rats (after one outlier removed from the obese + TT group), *n* = 3–4. **E** Bodyweight as a function of time of lean (*n* = 22) and obese + TT (*n* = 20) Zucker rats. Error bars are within the symbols. **F** Bodyweight at time of experimentation, **G** Bodyweight gain over testosterone injection period (final four weeks before experimentation), *n* = 16–22. Statistical significance evaluated with Student’s unpaired t-test and results shown as mean ± SEM. *p < 0.05, ***p < 0.001, ns = not significant

**Fig. 6:**
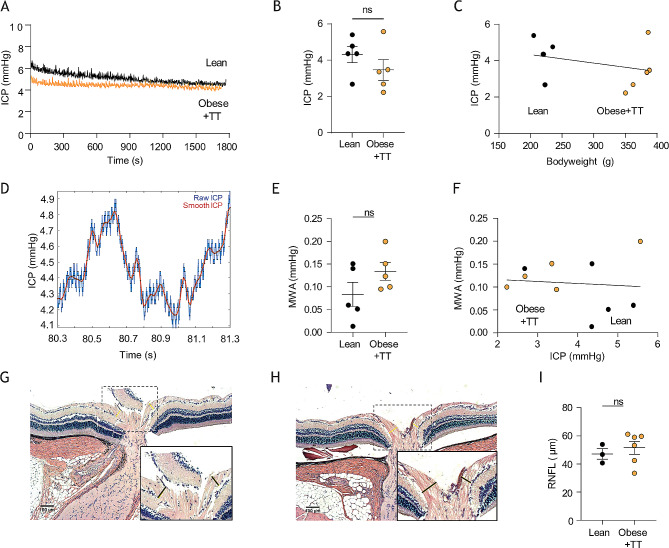
Intracranial pressure of testosterone-treated obese Zucker rats. **A** representative intracranial pressure (ICP) traces from lean and testosterone-treated obese (Obese + TT) Zucker rats with the final 15 min recordings quantified in **B**, *n* = 5 of each. **C** Correlation analysis of ICP as a function of bodyweight in the tested Zucker rats. **D** Representative ICP trace with “raw” (blue line) and “smoothed” (red line) signals, the latter used to calculate the mean wave amplitude (MWA), represented in **E** (*n* = 5 of each). **F** Correlation analysis of MWA as a function of the ICP, *n* = 10, *n*R^2^ = 0.01, *p* = 0.80. Histological preparations of lean (**G**) and testosterone-treated obese (**H**) rat eyes for quantification of the retinal nerve fiber layer (RNFL) thickness (indicated with black bars, see inset images) quantified in **I** (*n* = 6 obese + TT and *n* = 3 lean Zucker rats). Statistical significance evaluated with Student’s unpaired t-test or simple linear regression and results shown as mean ± SEM, ns = not significant

To determine whether rodent obesity of the present caliber caused the hormonal androgen disturbances detected in IIH patients [36], we performed liquid chromatography-mass spectrometry (LC-MS) analysis on CSF extracted from obese and lean rats. Androgen profiling revealed similar CSF testosterone levels of the two cohorts (1.92 ± 0.05 nmol/l, *n* = 4 in the obese rats vs. 1.88 ± 0.03 nmol/l, *n* = 3 in the lean rats, *p* = 0.62, Fig. 5A), see Additional file 3A for the entire androgen panel. Of note, as observed in IIH patients [36], obese Zucker rats displayed significantly elevated CSF androstenedione levels relative to lean counterparts (1.14 ± 0.20 nmol/l, *n* = 4 in the obese rats vs. 0.49 ± 0.04 nmol/l, *n* = 3 in the lean rats, *p* < 0.05, Fig. 5B). With these data suggesting that the testosterone elevation observed in obese female IIH patients is not recapitulated in the obese Zucker rats, we treated the obese Zucker rats with adjuvant testosterone for four weeks prior to experimentation. This treatment doubled the CSF testosterone level in the testosterone-treated (TT) obese Zucker rats (4.15 ± 0.76 nmol/l, *n* = 3 in the obese + TT rats vs. 1.72 ± 0.16 nmol/l, *n* = 4 in the lean control rats, *p* < 0.05, Fig. 5C). Obese + TT Zucker rats continued to display elevated CSF androstenedione levels (0.97 ± 0.03 nmol/l, *n* = 3 in the obese + TT rats vs. 0.67 ± 0.05 nmol/l, *n* = 4 in the lean control rats, *p* < 0.01, Fig. 5D), see Additional file 3B for the entire androgen panel. In addition, the obese + TT rats were heavier than their lean control counterparts (373 ± 5 g, *n* = 20 for obese + TT rats vs. 211 ± 13 g, *n* = 22 for lean control rats, *p* < 0.001, Fig. 5E-F), and gained weight faster than obese Zucker rats not treated with testosterone (23.0 ± 1.2% bodyweight increase in the final 4 weeks, *n* = 20 for the obese + TT rats vs. 10.5 ± 0.8% bodyweight increase in final 4 weeks, *n* = 16, for the obese rats from Fig. 1, *p* < 0.001, Fig. 5G). Nevertheless, the ICP was unaffected in the testosterone-treated obese rats (3.46 ± 0.57 mmHg in obese + TT rats, *n* = 5 vs. 4.31 ± 0.45 mmHg in lean control rats, *n* = 5, *p* = 0.28, Fig. 6A-B) with no correlation between bodyweight and ICP (*n* = 10, R^2^ = 0.10, *p* = 0.37, Fig. 6C). ICP waveform analysis revealed similar mean wave amplitudes in the two rodent cohorts (0.13 ± 0.02 mmHg, *n* = 5 in the obese + TT rats vs. 0.08 ± 0.02 mmHg, *n* = 5 in the lean control rats, *p* = 0.16, Fig. 6D-E) and showed no significant correlation with ICP (*n* = 10, R^2^ = 0.01, *p* = 0.80, Fig. 6F). Obese Zucker rats treated with adjuvant testosterone therefore did not present with the elevated ICP characteristic of IIH patients. To determine if these rats displayed ophthalmological phenotypes common to IIH patients [63], peripapillary retinal nerve fiber layer (RNFL) thickness was measured using histological sections of lean rats vs. obese rats treated with testosterone (Fig. 6G-H). This analysis revealed that the optic nerve head in both groups appeared normal without any pathology or signs of papilledema and with no significant difference in RNFL thickness between the two groups of rats (51.5 ± 4.6 μm, *n* = 6 in the obese + TT rats vs. 47.2 ± 3.8 μm, *n* = 3 in the lean control rats, *p* = 0.57, Fig. 6I).

### Testosterone-treatment of obese Zucker rats increases their CSF secretion rate

**Fig. 7:**
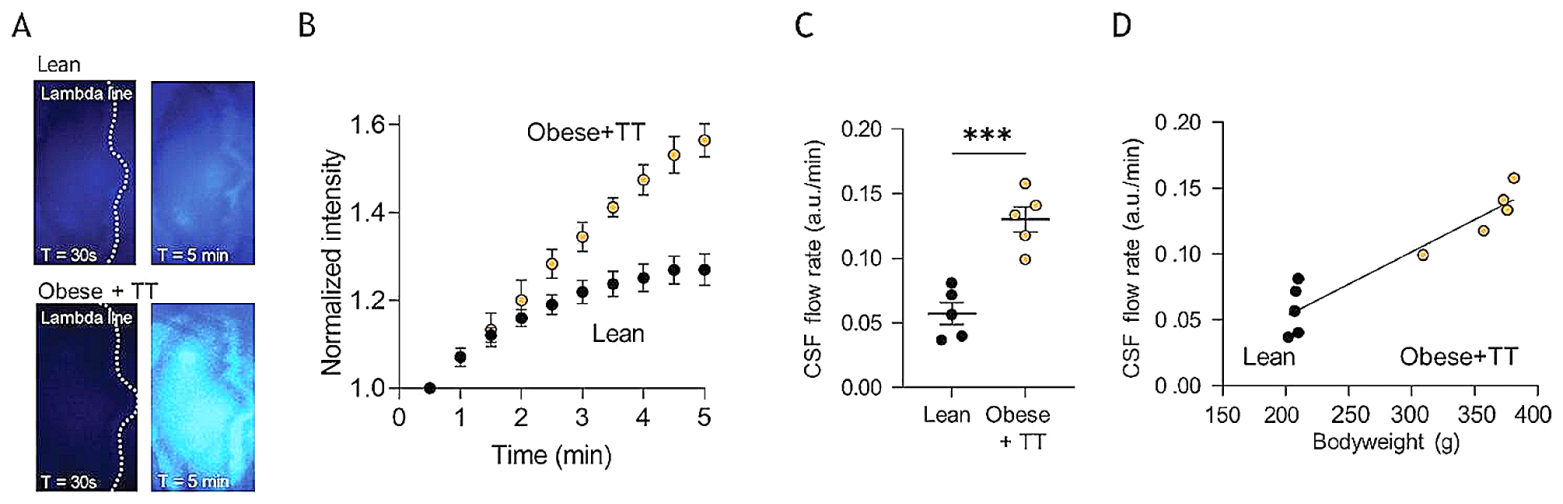
CSF flow determination in lean and testosterone treated obese Zucker rats. **A** Representative images of lean (top) and testosterone-treated obese (obese + TT, bottom) rats after (t = 30 s, left panels and t = 5 min, right panels) injection of IRDye 800CW carboxylate dye (superimposed pseudo-color). The dotted lines represent the lambda line, and image areas represent the area of dye quantification. **B** The dye intensity normalized to that obtained in the first image and plotted as a function of time, *n* = 5 of each group. **C** Quantification of the dye intensity as a function of time (the flow rate) determined from linear regression in **B** over the 5 min time window, *n* = 5 of each group. **D** Correlation of the CSF flow rate as a function of bodyweight, *n* = 5. Statistical significance evaluated with Student’s unpaired t-test or simple linear regression and results shown as mean ± SEM. ***p < 0.001

To determine whether adjuvant testosterone-treatment modulates the CSF secretion rate, we quantified the relative CSF flow rate in the testosterone-treated obese rats versus their lean control counterparts with LI-COR live imaging. This swift technique relies on quantification of caudal flow of a fluorescent probe delivered into the lateral ventricle of the anesthetized rat and aligns with CSF secretion patterns obtained with the ventriculo-cisternal perfusion assay [38, 39, 64], Fig. 7A-B, and allows testing of the rats in close sequence on the exact day of completion of the four week testosterone-treatment period. These experiments revealed an increased CSF flow in the testosterone-treated obese rats (0.13 ± 0.01 a.u./min in the obese + TT rats, *n* = 5 vs. 0.06 ± 0.01 a.u./min in the lean control rats, *n* = 5, *p* < 0.001, Fig. 7C), which displayed correlation to the rat bodyweight (*n* = 10, R^2^ = 0.89, *p* < 0.001, Fig. 7D).

### Transcriptional changes in choroid plexus with obesity combined with testosterone treatment

**Fig. 8:**
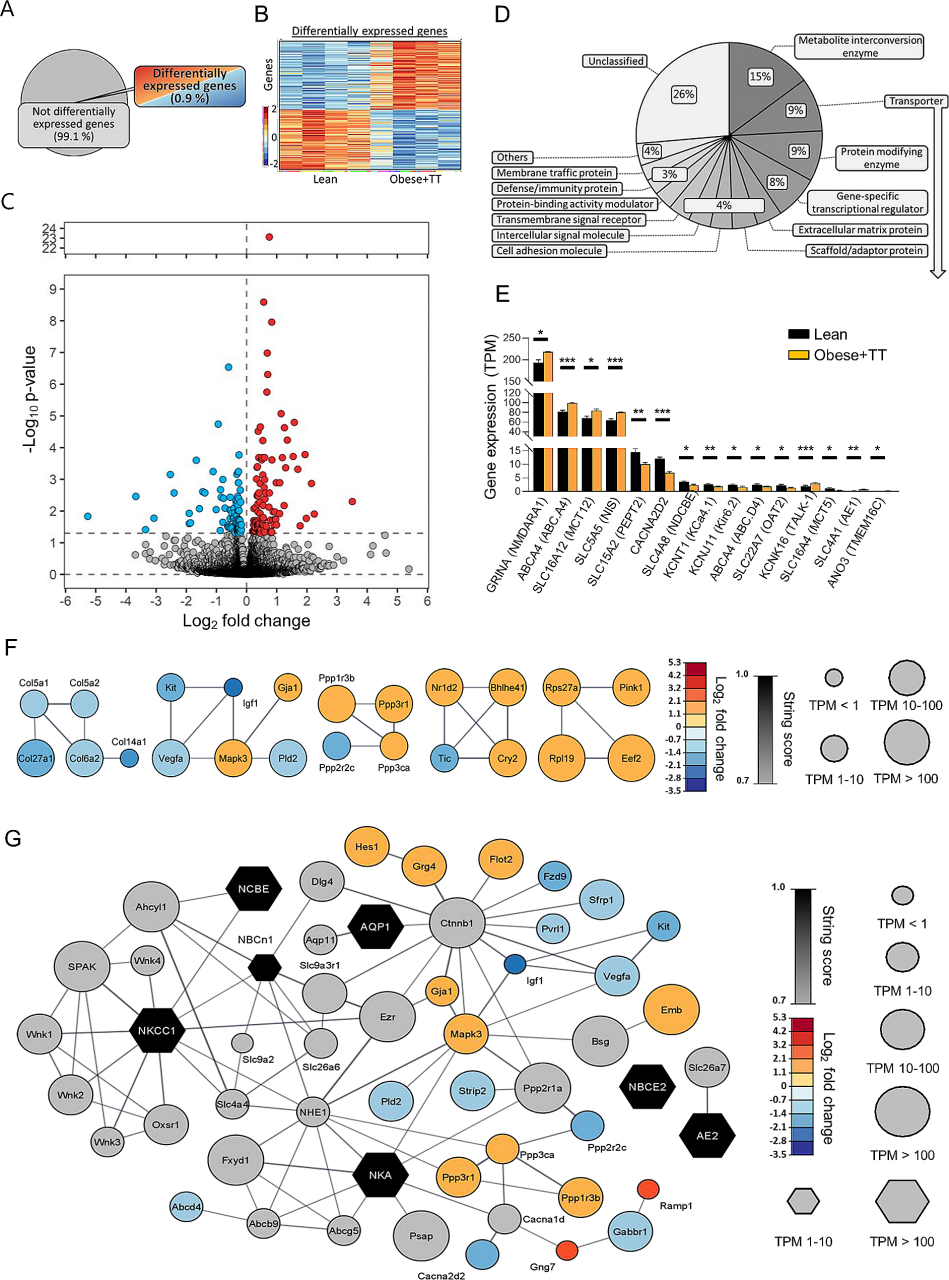
RNA expression in Zucker obese + TT rats. **A** illustrates a pie chart of the percentage differentially expressed genes. **B** A heatmap of the differentially expressed genes in the individual Zucker rats, coloured based on the Z-score of normalized fold changes (*n* = 4 of each). C Volcano plot of all transcribed genes detected in the choroid plexus of lean and obese + TT Zucker rats with differentially expressed genes (*p* < 0.05) marked in red (upregulated) or blue (downregulated). **D** GO enrichment analysis of the protein class categories. **E** Bar chart of all differentially expressed transporters in lean vs. obese + TT Zucker rats. Bar height is expression levels in transcript per million (TPM) with significance levels (* = *p* < 0.05, ** = *p* < 0.01, and *** = *p* < 0.001). **F** Association protein network analysis of the highest order cluster of genes. The protein nodes are coloured based on log2FC (downregulated in blue) and the connecting lines coloured based on the string confidence score from 0.7 (grey) to 1.0 (black). Size of the nodes are based on expression levels in four different categories. **G** Association protein network analysis of transporters (black) implicated in CSF productions with their first order protein connections expressed in the choroid plexus (grey) together with up- and downregulated proteins from the differentially expression analysis of lean vs. obese + TT rats. The protein nodes are coloured based on log2FC (downregulated in blue) and the connecting lines coloured based on the string confidence score from 0.7 (grey) to 1.0 (black). Size of the nodes are based on expression levels in four different categories

To determine if the elevated CSF secretion rate originated in altered expression of choroid plexus transporters supporting the CSF secretion apparatus, we performed RNAseq on choroid plexus extracted from testosterone-treated obese rats and their lean control counterparts. Of the 21,722 expressed genes detected in the testosterone-treated obese Zucker rat choroid plexus (Additional file 4, Sheet 1), 202 genes (0.9%) were differentially expressed (Fig. 8A). A heatmap illustrated approximately evenly distributed up- and downregulated genes (Fig. 8B), with a Volcano plot demonstrating 108 upregulated genes and 94 downregulated genes (Fig. 8C and Additional file 4, Sheet 2). We employed GO enrichment analysis of the 202 differentially expressed genes, where 134 genes could be categorized in protein classes to reveal the functional categories of the differentially expressed genes. These transcribed genes were dispersed in 12 different protein categories, including a pool of ‘others’ containing 9 categories with less than 4 genes assigned to these, and genes unassigned to any protein category labelled ‘unclassified’ (Fig. 8D). The most abundant categories included metabolite interconversion enzymes (15%), transporters (9%), protein-modifying enzymes (9%), and gene-specific transcriptional regulators (8%). With the transporters appearing as the second-highest category, we extracted the differentially expressed plasma membrane transporters within this category and organized them according to transcriptional abundance (Fig. 8E and Additional file 4, Sheet 3). The four most abundant differentially expressed transport mechanisms are all upregulated in the obese + TT rats, and include the monocarboxylate transporter MCT12 (*SLC16A12*), the sodium-dependent iodide symporter NIS (*SLC5A5*), the ABC transporter ABC.A4 (*ABCA4*), and the ionotropic glutamate receptor NMDARA1 (*GRINA*). Amongst the category of intermediate expressers (below 15 TPM), most genes are downregulated in the obese + TT rats (Fig. 8E). The differentially expressed genes were analyzed in an association network, which revealed only a few high order clusters (Fig. 8F). To include potential CSF regulatory pathways modulated by obesity and testosterone treatment, transporters known to be implicated in CSF production [65] were analyzed in association network with their first order connection, and differentially expressed genes were merged with this network. Figure 8G demonstrates the resulting network, which revealed obesity + TT-mediated transcriptional regulation of potential indirect candidate modulators of CSF secretion, e.g. kinases (e.g., MAPK3), phosphatases (e.g., PPP3r1 and PPP1r3b), phospholipases, (e.g., PLD2), and proteins involved in tethering of receptors (e.g., FLOT2).

### Testosterone-treated obese rats maintain their total brain fluid content but with altered fluid distribution

**Fig. 9:**
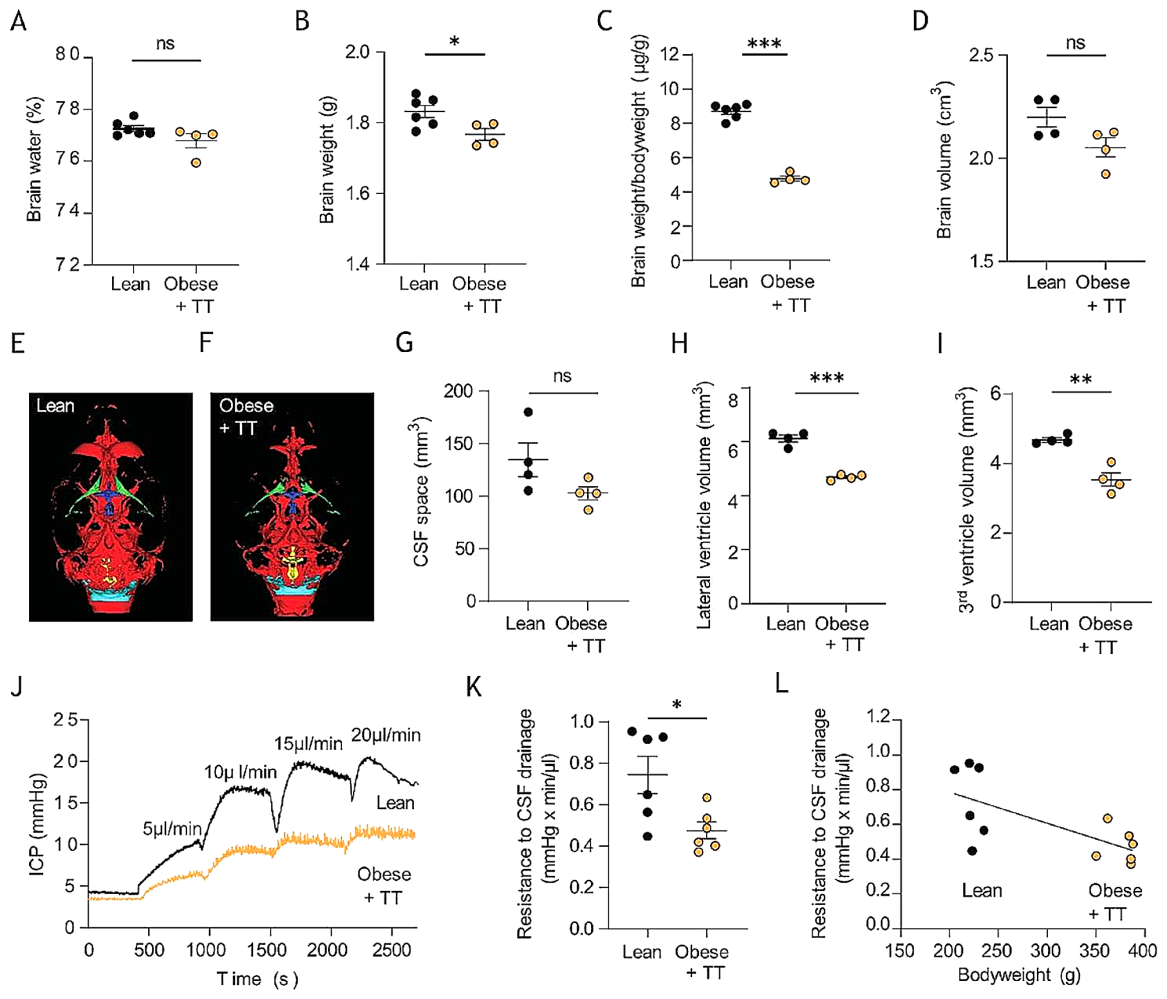
MRI analysis of CSF spaces and resistance to CSF drainage in testosterone-treated Zucker rats. **A** Brain water content in lean and testosterone-treated obese (obese + TT) Zucker rats, *n* = 4–6. **B** Brain weight and **C** relative brain weight of lean and obese Zucker rats, *n* = 4–6. **D** Brain volume obtained from the MRI scans, *n* = 4 of each. **E-F** Representative T_2_ MRI images from a lean (**E**) and an obese + TT (**F**) Zucker rats displaying total CSF spaces illustrated in green (lateral ventricles), dark blue (3^rd^ ventricle), yellow (4^th^ ventricle) or light blue (cisterna magna). **G** Total CSF space, **H** Lateral ventricle volume, and **I** 3^rd^ ventricle volume obtained from the MRI scans, *n* = 4 of each. **J** representative ICP traces of lean and obese + TT Zucker rats undergoing resistance to CSF drainage experiments by infusion of aCSF at the rates indicated in the figure. **K** Resistance to CSF drainage obtained in lean and obese + TT Zucker rats, *n* = 6 of each group. **L** Correlation analysis of the resistance to CSF drainage as a function of bodyweight, *n* = 12. Statistical significance evaluated with Student’s unpaired t-test or simple linear regression and results shown as mean ± SEM. *p < 0.05, **p < 0.01, ***p < 0.001, ns = not significant

The increased CSF secretion rate was not reflected in the brain water percentage, which did not differ between the two cohorts (77.3 ± 0.1%, *n* = 4 in the obese + TT rats vs. 76.8 ± 0.1%, *n* = 6 in lean control rats, *p* = 0.34, Fig. 9A), although the brain mass was significantly lower in the testosterone-treated obese rats, whether determined as total brain weight (1.77 ± 0.02 g, *n* = 4 in the obese + TT rats vs. 1.83 ± 0.02 g, *n* = 6 in the lean control rats, *p* < 0.05, Fig. 9B) or relative to bodyweight (4.77 ± 0.15 µg/g, *n* = 4 in the obese + TT rats vs. 8.68 ± 0.18 µg/g, *n* = 6 in the lean control rats, *p* < 0.001, Fig. 9C). Total brain volume in these rats displayed a tendency towards a reduction in the testosterone-treated obese rats (2.05 ± 0.05 cm^3^, *n* = 4 in the obese + TT rats vs. 2.20 ± 0.05 cm^3^, *n* = 4 in the lean control rats, *p* = 0.071, Fig. 9D), as quantified by MRI (Fig. 9E-F). MRI quantification was employed to determine whether the brain water dispersed differently in the testosterone-treated obese rats versus the lean control rats (Fig. 9E-F). The combined CSF space volume was similar (103 ± 13 mm^3^, *n* = 4 in the obese + TT rats vs. 135 ± 32 mm^3^, *n* = 4 in the lean control rats, *p* = 0.115, Fig. 9G). Curiously, the testosterone-treated obese rats had a reduced ventricular size in both the lateral ventricles (4.68 ± 0.05 mm^3^, *n* = 4 in the obese + TT rats vs. 6.11 ± 0.13 mm^3^, *n* = 4 in the lean control rats, *p* < 0.001, Fig. 9H) and the third ventricle (3.53 ± 0.19 mm^3^, *n* = 4 in the obese + TT rats vs. 4.68 ± 0.07 mm^3^, *n* = 4 in the lean control rats, *p* = 0.789, Fig. 9I; see Additional file 1 for quantification of all CSF spaces).

#### Testosterone-treated obese rats maintain their total brain fluid content via elevated CSF drainage

To explain the seeming hydrodynamic contradiction of elevated CSF secretion rate without brain fluid accumulation, we assessed the efficiency of CSF drainage from the cranial compartment. This technical approach relies on continuous ICP measurements during bouts of increasing infusion of artificial CSF, which allows for calculation of the CSF outflow resistance (R_out_) [43], Fig. 9J. These data revealed significantly lower CSF outflow resistance in testosterone-treated obese Zucker rats relative to lean controls (0.47 ± 0.04 mmHg × min/µl, *n* = 6 in obese + TT rats vs. 0.74 ± 0.09 mmHg × min/µl, *n* = 6 in lean control rats, *p* < 0.05, Fig. 9K). CSF outflow resistance correlated inversely with increased bodyweight (*n* = 12, R^2^ = 0.467, *p* < 0.05, Fig. 9L), which may account for lack of ICP elevation and total brain water accumulation despite increased CSF secretion in the testosterone-treated obese Zucker rats.

## Discussion

We here demonstrate that the marked obesity observed in a genetic rat model of obesity does not promote the elevated ICP characteristic of IIH patients, and was not associated with alterations in CSF dynamics or choroid plexus transcriptional profile. However, the rodent CSF secretion rate increased vastly in combination with adjuvant testosterone treatment designed to mimic the androgen excess observed in IIH patients [36].

IIH has been a recognized pathology since the 19th century [66, 67], but its underlying etilogy has remained unresolved. The associated elevated ICP suggests an uncompensated increase in the intracranial volume of any of the brain fluids, i.e., CSF, interstitial fluid (ISF), or blood. The latter has been proposed to increase with stenosis-induced increased central venous blood pressure [27, 28], although stenosis may arise secondarily to elevated ICP rather than being the underlying cause [68–70] and does not appear to correlate with the clinical course of the disease [71, 72]. Elevated CSF volume in IIH patients has been proposed [24–26], although not represented with the ventriculomegaly observed in hydrocephalus patients. The CSF drainage capacity is usually reduced [21–23], and the CSF may be redistributed to the subarachnoid space [73] or to the brain parenchyma, where it may promote diffuse brain edema and/or slit ventricles [30, 74]. These features, however, do not appear to be obligatory features of IIH patients [30, 73–77], leaving disturbed CSF dynamics as the underlying etiology unresolved.

To that end, we employed a female rat model of obesity to determine different aspects of CSF flow. We previously demonstrated that rats fed a high-fat diet became overweight and presented with an elevated ICP and reduced CSF drainage capacity [34], aligned with other studies also illustrating various other IIH features, including papilledema [33, 78]. The extent of the ICP elevation in these high-fat diet-fed rats, however, did not recapitulate the minimum doubling of opening lumbar pressure detected in IIH patients [79], possibly because of the limited weight gain observed in this cohort. We therefore here employed the Zucker rats, which with their deficient leptin receptor do not sense satiation, engage in hyperphagia on the normal chow, and become grossly obese (human BMI of roughly 32 after conversion of the rat BMI compared to roughly 20 for the lean Zucker rats). Despite the nearly doubling of their bodyweight, these rats did not present with elevated ICP, nor disturbed mean wave amplitude, and with no correlation between bodyweight and ICP. This finding contrasts an earlier one of its kind, performed with repeated ICP measurements in the same rat [80], but contradicts the notion that obesity, in itself, directly leads to the elevated intracranial pressure in rats that is characteristic of IIH in patients. We cannot rule out that rat age and development cycle does not mimic that of humans, and the 16 week old rats, approximately equal to 11 human years, may be on the pre-IIH stage of IIH pathophysiology in human patients [81]. In addition, the 16 weeks of hyperphagia represents a shorter window, than that experienced in IIH patients, in which aspects of obesity can exert its effect. Obesity-induced neuroendocrine imbalances and metabolic inflammation, which may be present in IIH patients, could therefore be absent from this rat cohort at this early time point [81]. Alternatively, one could speculate that the high sugar/high fat diet employed in the earlier studies on rats becoming obese on such ‘Western diet’ [33, 34, 78] could modulate the ICP in ways that are currently unknown, but absent in the Zucker rats that become obese on regular chow. The diet on which rats, and potentially humans, become obese on, may thus have direct implications on CSF drainage and possibly other ICP-related physiological aspects including blood flow. The obese Zucker rats, accordingly, had minor choroid plexus transcriptional changes, undisturbed CSF secretion rate and similar brain water content and distribution as compared to those of the lean Zucker rats. The CSF secretion rate can be measured with different technological approaches, which have advantages and disadvantages. We here employ the ventriculo-cisternal perfusion assay, which has been the gold standard for nearly a century [45, 65, 82, 83] and the LICOR-based live imaging of ventricularly-delivered fluorophore [34, 38]. The latter has the advantage of being a swift and direct measure of CSF flow in the ventricles, with easy inclusion of intraventricular inhibitors, and the disadvantage of only procuring relative CSF secretion rates, i.e. ideal for comparing two groups of animals as in the present study. The former has the advantage i) that the animals can be mechanically ventilated during the experimental procedure, which is essential for keeping the vascular parameters within physiological ranges and thus ensure optimal CSF secretion, ii) CSF secretion is quantified based on the contribution of all four choroid plexuses, and iii) one can readily and reliably introduce inhibitors into the ventricular compartment with the perfusate. The effect of inhibitors and activators of CSF secretion is identical in direct comparisons of these two techniques [38, 39, 64]. The ventriculo-cisternal perfusion technique has been questioned due to a suspicion of the diluted indicator diffusing into the brain parenchyma [84] of which we detected no sign [39], and due to lack of control with infusate temperature and osmolarity [84], which we secure with an in-line heater placed just prior to ventricular entry of the osmolarity-matched and gas-equilibrated artificial CSF [38, 39]. Although the absolute values of CSF secretion may or may not be accurately reflected in the ventriculo-cisternal perfusion assay, we here seek relative changes in two groups of rats. The so-called ‘direct method’, of CSF secretion quantification [85] does not allow the rodents to be ventilated (due to the required head angle), which may lower the CSF secretion rate. The ‘direct method’ does not include the contribution from the 4^th^ and largest choroid plexus (due to the oil-based block of the Aqueduct of Sylvius [85]), which will approximately halve the CSF secretion rate. Lastly, ventricular delivery of inhibitors lack accuracy as additional fluid is introduced and subsequently subtracted from the CSF exit [40, 85]. Inhibitor concentrations 50–100 fold higher than usually employed are thus necessary to obtain a reduction in CSF secretion [40, 85].

Obesity *in itself* does not appear to directly cause elevated ICP in humans [86] or rats (this study). IIH patients are not all obese [7, 86] and, accordingly, present with a range of alterations in various biomarkers that could indirectly modulate CSF dynamics [86]. Chief amongst these is the altered androgen profile detected in female IIH patients, who present with elevated testosterone and androstenedione levels in their CSF compared to obese non-IIH subjects [36]. Disturbed androgen profile may, on a whole, contribute to IIH pathogenesis as evidenced in the testosterone therapy employed in female to male gender transition likely promoting IIH [87–89], and, oppositely, testosterone deficiency in men representing a risk factor for IIH [90]. Elevated CSF testosterone was not detected in the obese female Zucker rats, but androstenedione was elevated in the CSF of the obese Zucker cohort to a similar extent as in IIH patients [36]. Androstenedione is the hormonal precursor directly preceding testosterone formation and may represent initial stages of testosterone accumulation that were not detectable in the obese Zucker rats at the employed experimental timeline. However, the rats were not controlled for their estrous cycle in this study, which may introduce cycle-dependent changes in choroid plexus that may mask testosterone-mediated changes in functionality and is thus considered a limitation to the study. Nevertheless, we previously demonstrated that adjuvant testosterone treatment of naïve lean rats caused elevated ICP and increased rates of CSF secretion, in part due to elevated activity of a choroid plexus transport protein, the Na^+^, K^+^, 2Cl^−^ cotransporter, NKCC1, [34] known to be involved in CSF secretion [38, 39, 91]. 4-week adjuvant testosterone treatment of obese female Zucker rats caused a vast increase in bodyweight and an approximate doubling of the CSF testosterone, similar to that observed in the patient cohort [36] and in the lean rats treated with adjuvant testosterone [34]. These rats now recapitulated two of the cardinal features of the IIH patients; obesity and elevated testosterone, and, importantly, demonstrated a robust increase in CSF secretion rate. Androgens act on the androgen receptor, expressed in the rat choroid plexus [92], and are known to induce activity of the SPS1-related proline/alanine-rich kinase (SPAK), highly expressed in the choroid plexus [92], and demonstrated as an activator of NKCC1 and thus CSF secretion [34, 40, 64, 93]. This coupling between elevated CSF testosterone and choroid plexus transport activity could provide a mechanistic link between the androgen tone observed in IIH patients and the disturbed brain fluid dynamics that may contribute to the elevated ICP in this patient group. Curiously, the higher CSF secretion could not be directly assigned to transcriptomic changes of transporters known to be involved in CSF secretion. However, network analysis of the differentially expressed genes merged with the association network of the first order connections of this subset of choroid plexus transporters, demonstrated potential regulatory factors, i.e. kinases, phosphatases, and phospholipases, modulated by obesity and adjuvant testosterone that could indirectly affect the rate of CSF secretion, although none of these have been investigated for their modulation of CSF secretion. However, as the RNAseq was performed on choroid plexus obtained from rats following the same treatment as those undergoing physiological experiments, these gene changes could have been present from birth. In addition, two of the upregulated cotransporters, the monocarboxylate transporter MCT12 and the Na^+^-dependent iodide transporter NIS, belong to families of transport proteins known to transport water as an inherent feature of their transport cycle [94–96] and could thus– although never tested– be involved in CSF secretion by the choroid plexus. An elevated expression of these could therefore contribute to the elevated rate of CSF secretion observed in these rats.

The elevated rate of CSF secretion observed in the testosterone-treated obese rats did not associate with elevated brain water content, increased ICP, or papilledema. This lack of total brain fluid accumulation could originate in the elevated CSF drainage capacity detected in obese testosterone-treated Zucker rats. The increased drainage capacity would, accordingly, prevent elevation of ICP, which in turn would prevent pressure-related ophthalmological disturbances characteristic of IIH, like papilledema [1]. Such unexpected elevation in CSF drainage capacity would allow the increased CSF production to readily dissipate from the brain and thus prevent brain fluid accumulation and associated ICP elevation. IIH patients usually present with *reduced* drainage capacity, as observed in the increased CSF outflow resistance (R_out_) [21–23], possibly arising with prolonged obesity and associated fibrosis and astrogliosis of drainage pathways [29, 97]. Disturbance of the glia-neurovascular interface in IIH patients has been previously described, and has been associated with this obesity-related astrogliosis [71, 98]. These rats therefore do not recapitulate this important feature of reduced CSF drainage capacity, generally associated with IIH and also detected in rats raised on high fat diet [34, 78], which is considered a limitation to the study. Curiously, despite the undisturbed overall brain water content detected in the obese, testosterone-treated rats, the lateral and third ventricle volume was reduced compared to their lean control counterparts. These findings could suggest fluid penetration into the brain parenchyma, as has been suggested for IIH patients [30, 74] and/or diverted to the subarachnoid space [73] and could relate to the distinct lack of ventriculomegaly (sometimes even reported slit ventricles [99]) observed in IIH patients, despite their elevated CSF pressure. Such smaller brain volume could contribute to the excess CSF drainage capacity and lack of ICP elevation in the obese rats. Interestingly, the decreased brain weight and volume that we observed in these obese Zucker rats is consistent with decreased total and grey matter volumes observed in brains of obese human patients [100–102].

In conclusion, we demonstrate that in experimental rats, obesity itself– at least when achieved on regular chow instead of a high-fat diet does not lead to elevated ICP or disturbed fluid homeostasis, as is also the case in humans, where a range of co-factors appear to separate the IIH patients from age-, sex-, and BMI-matched control subjects [36, 86]. Combination of two cardinal features observed in female IIH patients; obesity and elevated CSF androgen tone, caused an increase in the rate of CSF secretion in these rats, which, however, did not lead to elevated ICP due to the elevated drainage capacity in the obese rats. If future clinical research detects this feature of testosterone-mediated elevated CSF flow rates in IIH patients, combined with the reduced CSF drainage capacity observed in this patient group, one could imagine– over prolonged time– that these two features together could contribute to the elevated ICP characteristic of IIH. Androgen blockage and/or therapeutic modulation of the CSF secretion machinery may thus, in the context of obesity, elucidate novel avenues for pharmaceutical therapy of IIH patients.

### Electronic supplementary material

Below is the link to the electronic supplementary material.

Supplementary Material 1

Supplementary Material 2

Supplementary Material 3

Supplementary Material 4

## Data Availability

The datasets used in the current study are available from the corresponding author on reasonable request. Raw RNAseq data are available at the NCBI GEO database with accession number GSE232814, https://www.ncbi.nlm.nih.gov/geo/query/acc.cgi?acc=GSE232814. Scripts and data analysis are available at: https://github.com/Sorennorge/MacAulayLab-RNAseq3-Zucker.
