# Supplementary material for: CSF hyperdynamics in rats mimicking the obesity and androgen excess characteristic of patients with idiopathic intracranial hypertension
Source: Fluids Barriers CNS. 2024 Jan 25;21:10. doi: 10.1186/s12987-024-00511-1 (PMC10810013; doi:10.1186/s12987-024-00511-1)
Supplement: Supplementary file 1 — Supplementary Material 1 [file 12987_2024_511_MOESM1_ESM.pdf]

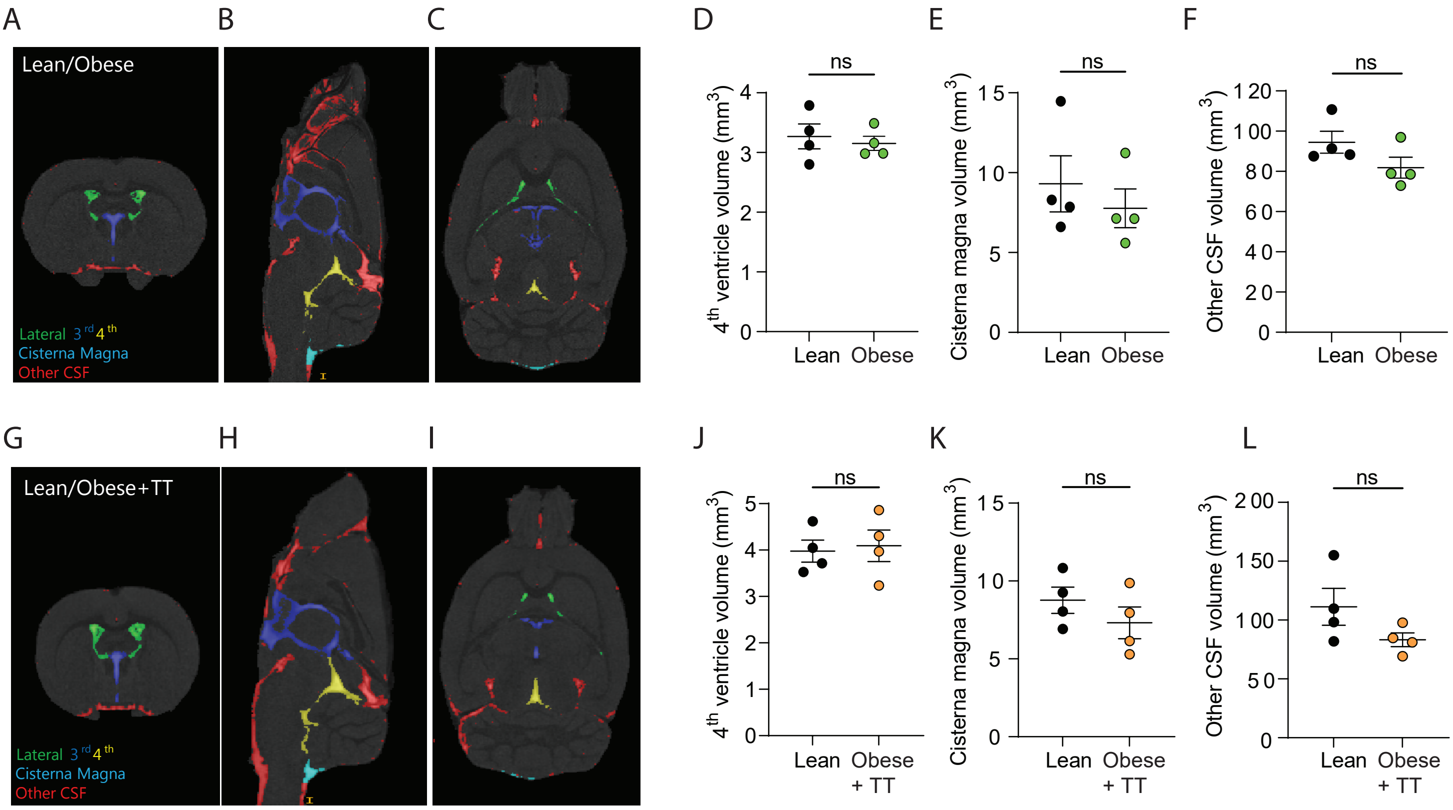

Additional file 1. T_2 MRI of Zucker rats brains: Defined CSF volumes in Zucker rat brains used for quantification of CSF subvolumes **A** Coronal, **B** sagittal and **C** axial cross sections of obese Zucker rat brains. Quantification of **D** fourth ventricle**, E** cisterna magna and **F** other (excluding all other specifically quantified volumes) CSF volumes in Zucker lean and obese rats (n=4 of each group). **G** Coronal, **H** sagittal and **I** axial cross sections of Zucker rat brains from vehicle/testosterone treated cohort . Quantification of **D** fourth ventricle, **E** cisterna magna and **F** other (excluding all other specifically quantified volumes) CSF volumes in Zucker lean and testosterone-treated obese (Obese+TT) rats (n=4 of each group). Statistical significance evaluated with Student's unpaired t-test. Results are shown as mean ± SEM ns = not significant.
