# Supplementary material for: CSF hyperdynamics in rats mimicking the obesity and androgen excess characteristic of patients with idiopathic intracranial hypertension
Source: Fluids Barriers CNS. 2024 Jan 25;21:10. doi: 10.1186/s12987-024-00511-1 (PMC10810013; doi:10.1186/s12987-024-00511-1)
Supplement: Supplementary file 3 — Supplementary Material 3 [file 12987_2024_511_MOESM3_ESM.pdf]

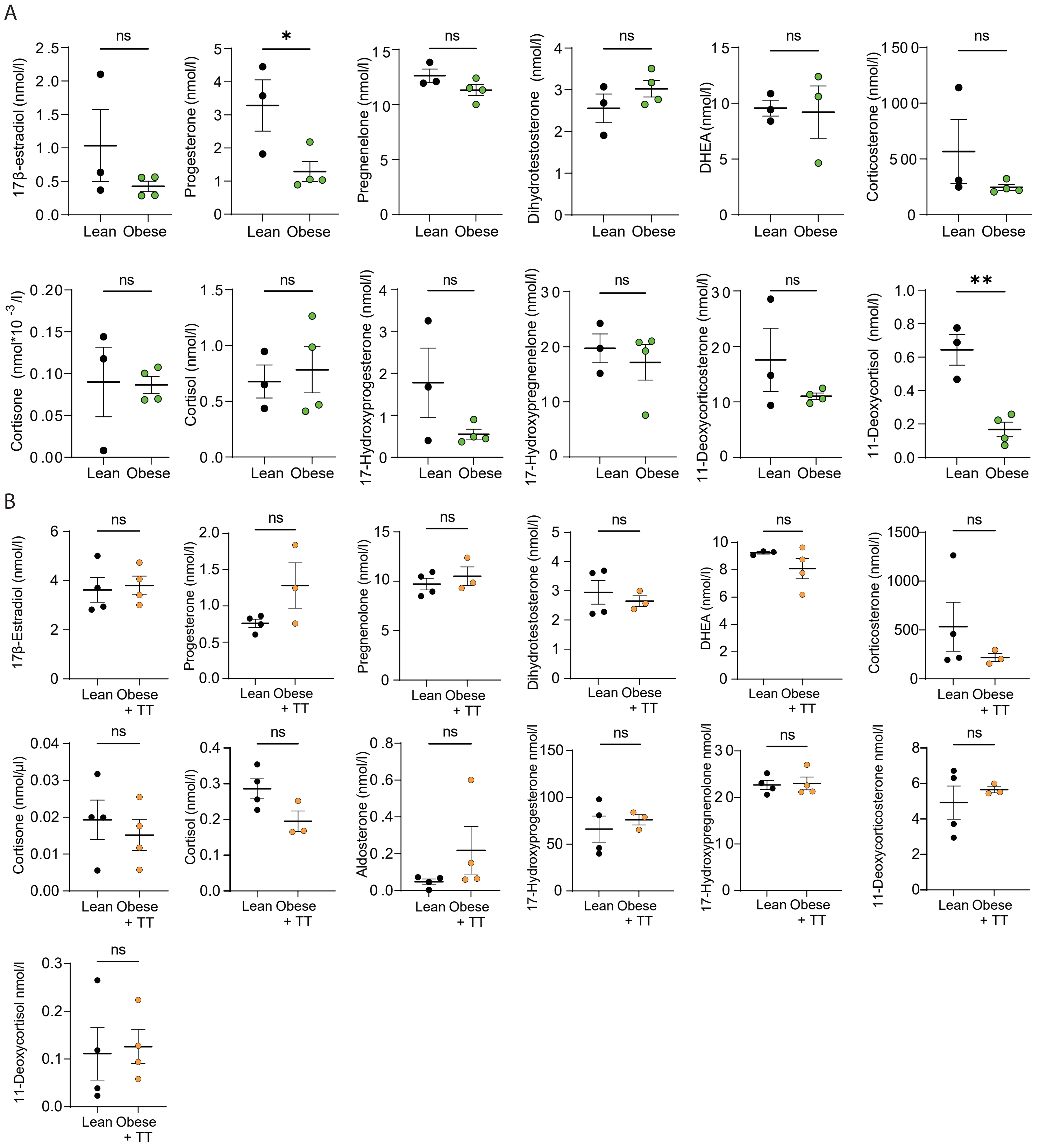

Additional file 3. Mass spectrometry analysis of CSF hormones: A CSF hormone levels in lean and obese Zucker rats. **B** CSF hormone levels in lean and testosterone-treated obese (obese+TT) Zucker rats Progesterone and 11-deoxycortisol are are significantly decreased in the CSF of obese Zucker rats. Statistical significance evaluated with Student's t-test. Results are shown as mean ± SEM. * P <0.05, ** P<0.01, ns = not significant.
